# Micro-Twinning in IN738LC Manufactured with Laser Powder Bed Fusion

**DOI:** 10.3390/ma16175918

**Published:** 2023-08-29

**Authors:** Sandra Megahed, Karl Michael Krämer, Christian Kontermann, Christoph Heinze, Annett Udoh, Stefan Weihe, Matthias Oechsner

**Affiliations:** 1 Chair and Institute for Materials Technology, Technical University of Darmstadt, Grafenstr. 2, 64283 Darmstadt, Germany; 2 Siemens Energy Global GmbH & Co. KG, Gas Services, Additive Manufacturing Technology, Innovation and Digitalisation, Huttenstr. 12, 10553 Berlin, Germany; 3 Materials Testing Institute, University of Stuttgart, Pfaffenwaldring 32, 70569 Stuttgart, Germany

**Keywords:** laser powder bed fusion, IN738LC, microstructure, creep, micro-twinning

## Abstract

Components manufactured with Metal Laser Powder Bed Fusion (PBF-LB/M) are built in a layerwise fashion. The PBF-LB/M build orientation affects grain morphology and orientation. Depending on the build orientation, microstructures from equiaxed to textured grains can develop. In the case of a textured microstructure, a clear anisotropy of the mechanical properties affecting short- and long-term mechanical properties can be observed, which must be considered in the component design. Within the scope of this study, the IN738LC tensile and creep properties of PBF-LB/M samples manufactured in 0° (perpendicular to build direction), 45° and 90° (parallel to build direction) build orientations were investigated. While the hot tensile results (at 850 °C) are as expected, where the tensile properties of the 45° build orientation lay between those of 0° and 90°, the creep results (performed at 850 °C and 200 MPa) of the 45° build orientation show the least time to rupture. This study discusses the microstructural reasoning behind the peculiar creep behavior of 45° oriented IN738LC samples and correlates the results to heat-treated microstructures and the solidification conditions of the PBF-LB/M process itself.

## 1. Introduction

Additive manufacturing creates objects using a sliced 3D CAD geometry [1,2,3]. The successive build-up of components increases the design freedom, enabling the manufacture of complex geometries using Metal Laser Powder Bed Fusion (PBF-LB/M) [4,5].

PBF-LB/M creep curves for IN738LC are scarce in the existing literature due to the extensive experiments required for conventionally manufactured materials combined with the many PBF-LB/M parameter combinations to be considered. Hence, the required creep sample number for PBF-LB/M is significantly higher compared to conventionally manufactured samples.

Depending on the build orientation, microstructures from equiaxed to textured grains can develop. Sanchez et al. investigated the IN718 creep behavior for 0° (perpendicular to build direction), 45° and 90° (parallel to build direction) build orientations [6]. It was shown that while the 45° build orientation lies within the bounds of 0° and 90°, the difference between 0° and 45° is small. In comparison to the low creep strains and times to rupture of the IN718 0° sample, the 90° sample showed a significantly higher creep strain and time to rupture with an extended tertiary creep stage [6].

For IN738LC, the creep strength of 0° samples (perpendicular to build direction) lies below the creep strength of 90° samples (parallel to build direction) [7]. Results for IN738LC 45° samples were first reported on in 2023 [8].

Microstructural creep deformation mechanisms in polycrystalline nickel superalloys are complex [9,10]. The deformation mechanism depends on load, microstructure and crystallography [11]. Kear et al. first introduced micro-twinning as a possible deformation mechanism [12]. Inhomogenous chemical distribution leads to differences in stacking fault structures, especially at temperatures ranging between 650 °C ≤ x ≤ 800 °C [10,13,14,15]. Barba et al. established a compositional dependency of micro-twin formation to segregations of Co, Cr, Nb, W and Ta [13,16]. A microstructural dependency has also been reported by Titus et al. [17]. Segregations of Co, Cr, W and Ta lead to Superlattice Intrinsic Stacking Faults (SISF) and Superlattice Extrinsic Stacking Faults (SESF) [17]. Micro-twins have been found preferentially at SISFs and SESFs within the γ’ phase [13,14,16,18].

Brittle material behavior has been found in relation to micro-twins [19]. Micro-twins lead to a dislocation pile-up, since the slip systems are limited to <111> bands. An abrupt change in Schmid factor has been reported on along the twin-parent interface [19]. These dislocation pile-ups increase stress concentrations, causing crack initiation and propagation along the twin and neighboring grain interface.

Due to the small sizes of micro-twins (usually between 7–12 atomic planes), they are often mistaken for deformation twins. The lack of understanding and confusion with deformation twins, often leads to inconsistent statements concerning the effect on ductility.

From the surveyed literature, it is apparent that only a limited number of papers study the creep behavior of PBF-LB/M materials. The general understanding of the creep performance of PBF-LB/M materials is therefore limited [20]. In this study, the creep properties of the 45° build orientation for IN738LC were found to be affected by micro-twins, the origin of which can be attributed to PBF-LB/M solidification conditions.

## 2. Materials and Methods

### 2.1. Materials

As a high-temperature material with promising mechanical properties for the gas industry, IN738LC is the investigated material in this study. The IN738LC powder used in this study, with the chemical composition shown in Table 1, has the following characteristics:

- Supplier: VDM Metals (Frankfurt, Germany);
- Gas atomized;
- The D_10_–D_90_ range corresponds to 22–49 µm;
- B, P, and S lie below 0.015 wt.%.;
- Mn and Fe lies below 0.05 wt.%.;
- Less than 0.3 wt.% Si are contained within the powder.

**Table 1:** Chemical composition of IN738LC powder in wt.%.

Al	C	Co	Cr	Mo	Nb	Ni	Ta	Ti	W	Zr
**3.7**	0.13	9	16.3	2	1.1	58.6	2	3.7	2.8	0.08

### 2.2. PBF-LB/M Manufacturing

Since the PBF-LB/M processing window is small, the processing conditions should be adjusted in order to increase mechanical properties while reducing internal defects (such as porosity) [21].

The details of sample dimensions and PBF-LB/M manufacturing are listed in Table 2. Three build orientations were considered: 0° (perpendicular to build direction), 45° (diagonal) and 90° (parallel to build direction).

### 2.3. Creep and Tensile Testing

The high-temperature properties of PBF-LB/M IN738LC must be characterized under the conditions of the intended application (i.e., gas industry).

**Figure 1:**
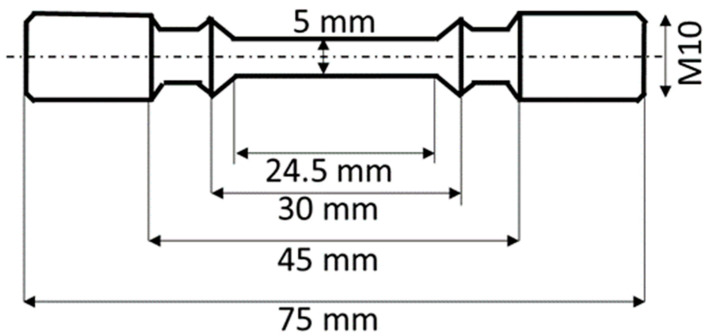
Creep and tensile sample geometry.

For creep and tensile testing, the PBF-LB/M cylinders were machined into the geometry shown in Figure 1.

Creep tests were carried out according to DIN EN ISO 204 [22] with the following parameters:

- Temperature: 850 °C;
- Applied stress: 200 MPa.

The tensile tests were carried out according to DIN EN ISO 7500-1 [23], with the following parameters:

- Temperature: 850 °C;
- Strain rate: 0.5%/min when ε < 1.5% (elastic regime);
- Strain rate: 5%/min when ε > 1.5% (plastic regime).

### 2.4. Relative Density and Microstructure

Internal defects negatively affect short- and long-term properties. Therefore, the relative density is characterized to determine whether the samples contain porosity or cracks.

As mentioned in the introduction, microstructural creep deformation mechanisms are complex. Therefore, microstructural analysis was carried out using scanning and transmission electron microscopy, electron back scatter diffraction and energy-dispersive X-ray to characterize the active deformation mechanisms, including micro-twinning.

#### 2.4.1. Relative Density

Samples were cross-sectioned, ground and polished using 1 µm diamond solution.

Using a light microscope (Leitz Aristomet, Wetzlar, Germany) five images (2 mm × 1.5 mm field) were taken at different locations per sample parallel and perpendicular to build direction. The images were converted to binary images using ImageJ (version 1.54). The relative density was determined based on image contrast.

#### 2.4.2. Scanning Electron Microscopy (SEM)

Microstructural analysis was carried out on a SEM (Zeiss Auriga, Oberkochen, Germany). For that purpose, samples were etched using V2A etchant. The microstructure parallel and perpendicular to build direction was investigated. Particular focus lies on grain size and twinning (twin density and twin thickness). For each SEM image, twins were counted and divided by the image area. The twin thickness was measured using ImageJ.

For phase analysis, grain boundary and Euler angles and Schmid factor analysis, Electron Back Scatter Diffraction (EBSD) and Energy dispersive X-ray (EDX) analysis were carried out on polished samples parallel to build direction at the center of the respective samples (distance from substrate plate 0°: ~8 mm; 45°: ~38 mm; 90°: ~40 mm).

#### 2.4.3. Transmission Electron Microscopy (TEM)

For further twin analysis, TEM analysis was necessary. Using spark erosion, a 3 mm cylinder was extracted from the samples and cut into discs. The thickness of the discs was reduced to 50–200 nm by grinding and electrochemical thinning. TEM analysis was carried out on an JEOL JEM 2000 FX (Freising, Germany).

### 2.5. Numerical Thermal Modelling

The solidification conditions during the PBF-LB/M process significantly affect the microstructure of samples. Thus, the thermal history of the manufactured samples is visualized and quantified using numerical models.

The estimation of the thermal history during the PBF-LB/M manufacturing process was carried out using OpenFOAM software (version 11) by solving Fourier’s law (see Equation (1)). Since the computational effort is high for numerical solutions reported in the literature [24,25,26,27,28,29], the following is assumed:

- The thermal model used in this study ignores melt pool dynamics and does not resolve scan strategy for the sake of reducing computational effort. Zielinksi et al. showed, that layer-wise thermal models are able to accurately describe the thermal history of parts, while also increasing computational speed [30].
- The volume energy density is applied to 100 layers at a time lasting for a duration corresponding to the build rate and the respective layer volume. After that, the heat source is switched off and allows for the induced heat to diffuse during the recoating time (10 s). Since a group of layers is heated at once, the recoating time is extended accordingly. Following the recoating time, the next 100 layers are exposed to the volume energy density. The process is repeated until the complete samples are numerically built.
- The boiling temperature (i.e., 3200 K) is set as the upper temperature limit.
- In the mushy zone (T_solidus_ to T_liquidus_), the solution is held constant until the material melts or solidifies.
- The substrate plate acts as a heat sink, with the substrate plate sides exchanging heat with a heat transfer coefficient of 10 $\frac{W}{m\cdot K\;}$, and the substrate plate top exchanges heat with a heat transfer coefficient of 5 $\frac{W}{m\cdot K\;}$.
- The properties ρ, *c_p_* and λ are temperature dependent and were taken from [31].

(1) $$\rho c_{p}\frac{dT}{dt}=\nabla \cdot \left(\lambda \nabla T\right)+Q_{v}$$

where:

- ρ is the density;
- *c_p_* is the specific heat;
- *T* is temperature;
- *t* is time;
- λ is thermal conductivity;
- *Q_v_* is volumetric heat source.

## 3. Results and Discussion

### 3.1. PBF-LB/M Microstructure

**Figure 2:**
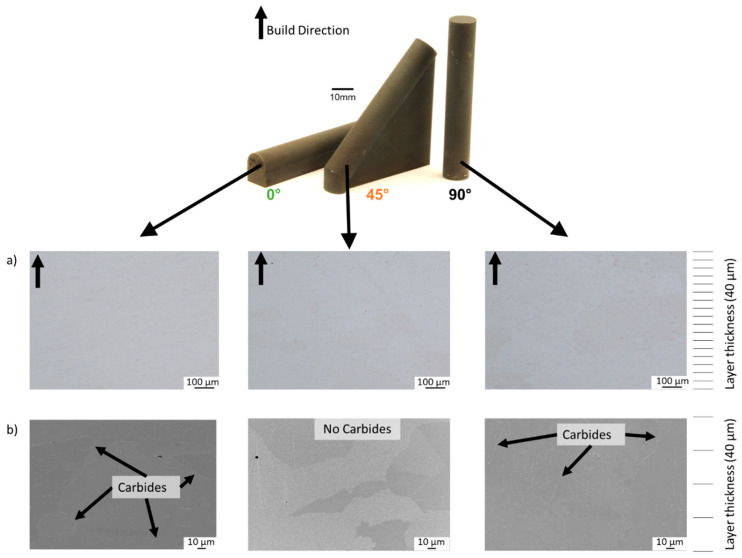
(**a**) IN738LC cross-sections parallel to build direction for all considered build orientations (0°, 45°, 90°); (**b**) microstructure parallel to build direction for all considered build orientations (0°, 45°, 90°).

The printed IN738LC samples are shown in Figure 2. The 45° and 0° samples were printed onto volume supports to avoid distortion caused by thermal stresses [32,33].

In Figure 2a, cross-sections of the considered build orientations (0°, 45° and 90°) can be seen. A relative density of 99.98% was determined based on contrast differences within the cross-sections. The determined relative density is in agreement with the literature [7,34,35,36].

The microstructure for all build orientations is shown in Figure 2b. The microstructure in the XZ-plane shows grains oriented parallel to the build direction, especially for the 90° build orientation. Considering the layer thickness used (i.e., 40 µm), the microstructure shows epitaxial grain growth over more than 5 layers. The scale on the right-hand side of Figure 2, indicates how many layers are resolved. While the 45° build orientation does not show any carbides, the 0° and 90° build orientations show carbides concentrating at the grain boundaries.

To determine the cause behind the microstructural difference, numerical modelling of the PBF-LB/M thermal conditions was carried out.

**Figure 3:**
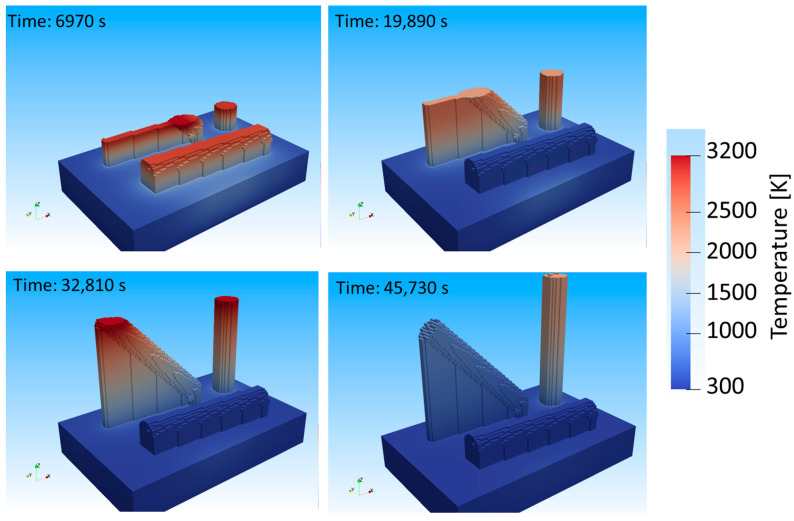
Temperature distribution during PBF-LB/M processing according to numerical model.

Figure 3 shows a sequence of images, visualizing the sample temperatures throughout the build process. The respective build job time is shown in the upper left corner of each image in seconds. After 6970 s, the 0° sample is almost completed. The upper layer generally reaches higher temperatures, close to boiling. The heat is dissipated downwards towards the substrate plate through the support structure. At 19,890 s, the 0° sample is concluded and has cooled down significantly as the laser continues to scan the 45° and 90° samples. The image at 32,810 s shows the temperature distribution after laser scanning, where the upper layers are cooling down from peak values seen during laser processing. After 45,730 s, all samples have been built and additional time has been modelled to capture the cool down prior to the removal of the substrate plate from the machine.

**Figure 4:**
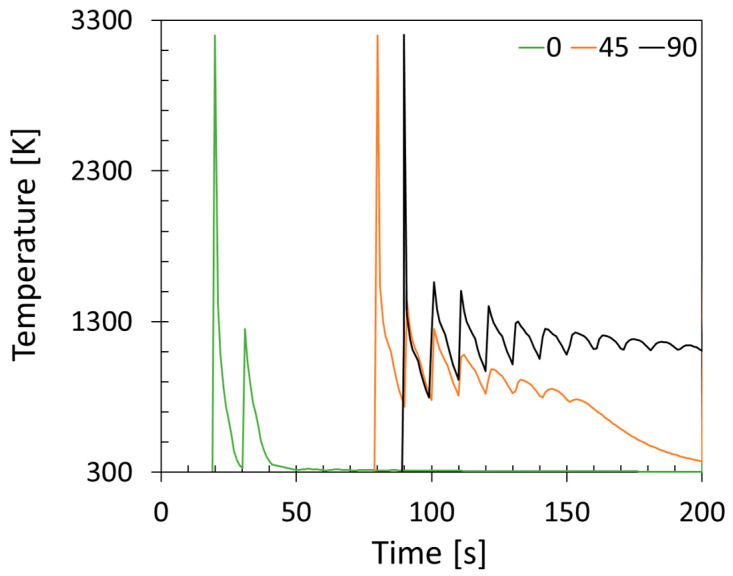
Numerically predicted temperature history in the center of each sample.

The temperature evolution at the center of each creep sample is shown in Figure 4. The peaks show the heating of 100 layers at once. As can be identified, the 90° sample reaches considerably higher temperatures compared to the 0° and 45° samples. This is explained by the difference in cross-sections between the build orientations. The 90° sample has the smallest cross-section of 133 mm^2^ between the sample and the substrate, whereas the 45° and 0° samples have cross-sections of 270 mm^2^ and 1500 mm^2^, respectively. The energy induced into the 90° sample is transferred into the substrate plate at a reduced rate compared to 0° and 45°, thereby retaining the heat within the sample causing an in situ heat treatment. The 45° and 0° build orientations experience faster cooling rates compared to the 90° sample.

While the 0° and 45° build orientations seem to show similar thermal histories, the 0° build orientation solidifies considerably faster compared to the 45°, due to the small build height (i.e., 16 mm). As can be seen in Figure 3 at 45,730 s, the 0° sample is much cooler compared to the 45° build orientation, which still retains some of the heat, while significantly less than the 90° build orientation.

The average grain sizes correlate well with the differing solidification conditions. The average grain size of the 0° build orientation corresponds to 108 ± 13 µm; for the 45° build orientation, 60 ± 7 µm; and for the 90°, 162 ± 15 µm. The 0° and 45° samples show significantly smaller grain sizes compared to the 90° sample, due to grain coarsening resulting from the in situ heat treatment of the 90° build orientation.

**Figure 5:**
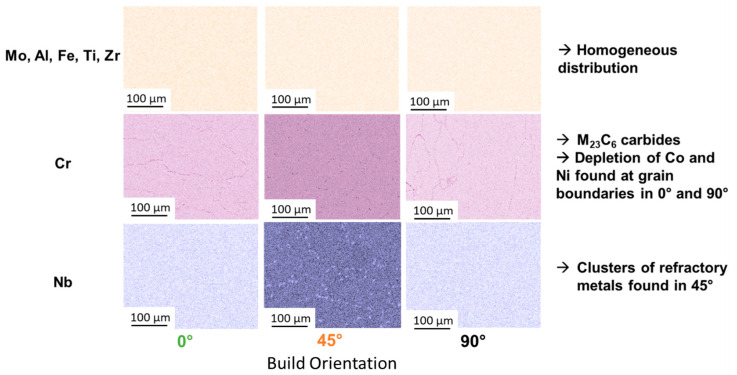
EDX elemental maps for Mo, Al, Fe, Ti, Zr, Cr and Nb in the 0°, 45° and 90° build orientation.

With the difference in solidification conditions, segregation behavior should also be considered. EDX mappings were determined to show how the elemental distribution is affected (see Figure 5). The 0° and 90° build orientation show Cr along the grain boundaries. Correlating the EDX maps to the SEM images in Figure 2b, these precipitates are Cr_23_C_6_-type carbides. The 45° build orientation does not show Cr segregations at grain boundaries (as expected, since no carbides were found in Figure 2b); however, clusters of Nb could be found. These Nb clusters were not found for the 0° and 90° samples. Nb is a refractory metal with a relatively large atom size (atom radius of 146 pm). This atom size requires more time/heat for diffusion. The fast solidification of the 0° sample does not allow enough time for Nb to diffuse and segregate, and the in situ heat treatment of the 90° sample homogenizes the elemental distribution. The 45° sample retains some of the heat, allowing enough time for Nb to segregate but not enough time for homogenization.

**Figure 6:**
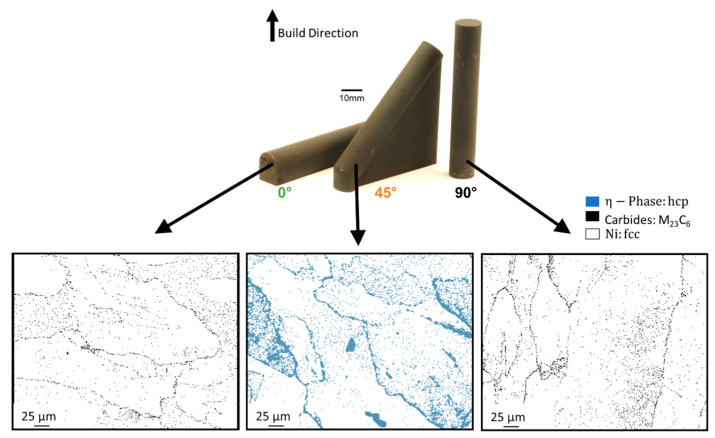
Phase identification at the center of the 0°, 45° and 90° build orientations.

EBSD-maps were used to identify the phases found in 0°, 45° and 90° build orientations at the respective sample center (see Figure 6). As expected, the 0° and 90° build orientations show a combination of face center cubic (fcc) phases (i.e., γ and $\gamma^{\prime }$) and carbides (as predicted by Cr-maps in Figure 5 and SEM images in Figure 2b). The 45° sample shows a combination of fcc phases and hexagonal close packed (hcp) phases.

The hcp-phase in IN738LC includes the η-phase (Ni_3_Ti or Ni_3_Ta). The η-phase forms when exposed to temperatures between 850 °C to 950 °C. At that temperature range, carbides decompose thereby enriching the grain boundaries with Ti and Ta. The $\gamma^{\prime }$-phase becomes unstable due to the Ti or Ta segregations and the η-phase forms [37]. Since the formation of the η-phase is dependent on the dissolution of carbides, this hcp phase forms at grain boundaries. The η-phase is known to increase strength and reduce ductility due to the incoherency of the hcp crystal structure with the γ-matrix [37,38].

The fast solidification of the 0° build orientation does not allow enough time for the η-phase to form, since thermal exposure is required for the decomposition of carbides to occur. The in situ heat treatment of the 90° build orientation homogenizes elemental distribution, hindering η-phase formation. The 45° sample, which retains temperatures of roughly 1000 K (compare Figure 3 at 45,730 s), allows for optimal η-phase formation conditions.

These microstructural differences lead to the assumption that mechanical properties are affected by the build orientation. The mechanical properties will be investigated and correlated to the microstructure in the following sub-sections.

### 3.2. PBF-LB/M Tensile Properties

**Figure 7:**
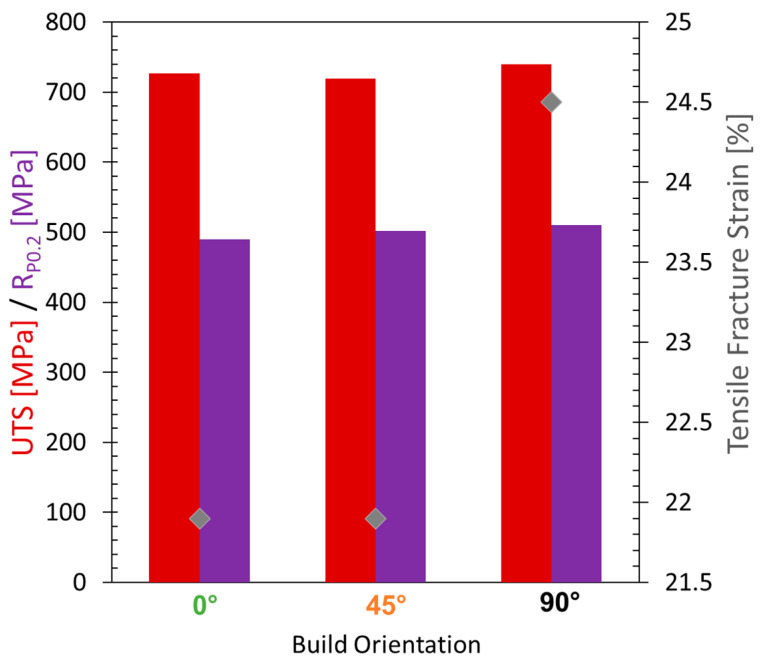
Overview of IN738LC tensile properties at 850 °C for the 0°, 45° and 90° build orientation.

Based on literature [7], it is expected that the 90° build orientation shows the largest ductility but lowest strengths compared to 0° and 45° build direction. The 45° build orientation is expected to lie within the bounds of the 0° and 90° results. An overview of the tensile properties determined in this study is shown in Figure 7. The yield strengths (R_P0.2_) and ultimate tensile strengths (UTS) of all build orientations vary less than 5%. The difference is therefore negligible. The 90° build orientation shows a ductility of 24.5%, the 0° and 45° build orientations show a lower ductility of 21.9%. These results correlate well with the numerical results, since the 0° and 45° samples show similar solidification conditions.

### 3.3. PBF-LB/M Creep Properties

**Figure 8:**
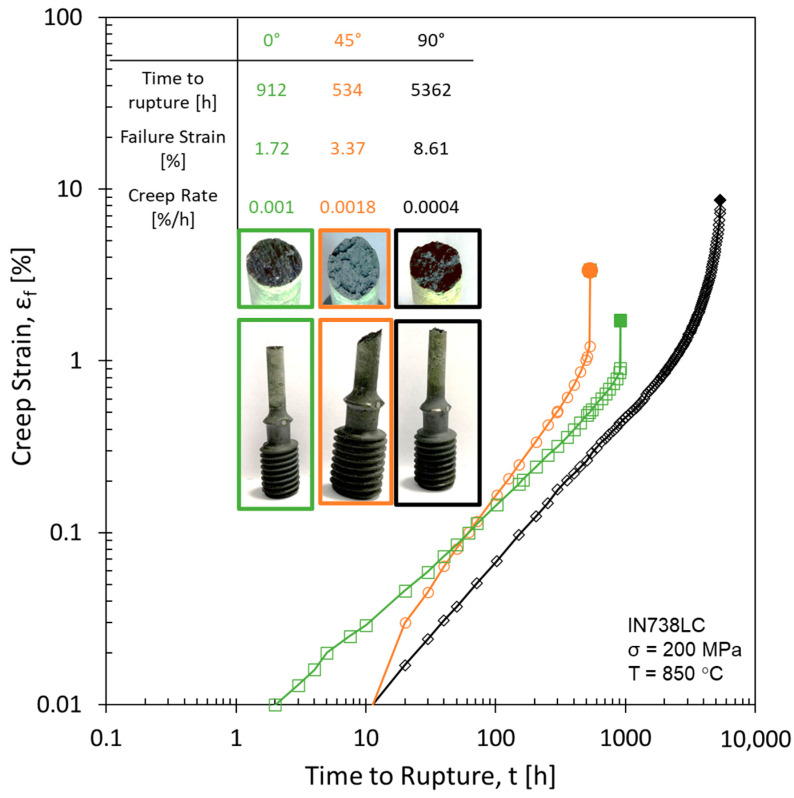
IN738LC creep results for 850 °C and 200 MPa.

The creep results are shown in Figure 8. As expected, the creep rupture strains increase from 0° (1.7%) to 45° (3.37%) to 90° (8.61%). The times to rupture increase from 45° (534 h) to 0° (912 h) to 90° (5362 h). While the 45° build orientation shows an expected creep rupture strain of 3.37%, the time to rupture is significantly smaller than expected (i.e., 534 h). As can be seen from the exact creep rate values listed in Figure 8, the minimum creep rates of the 0° and 90° build orientations are lower compared to the 45° build orientation. The reduced grain size and lack of carbides (see Figure 2b) in the 45° sample could facilitate grain boundary sliding increasing creep rate. In comparison, the carbides in the 0° and 90° build orientations hinder grain boundary motion reducing the creep rate.

Based on the creep test parameters used (850 °C and 200 MPa) and the deformation mechanism map (i.e., shear modulus vs. homologous temperature [39]) the expected primary deformation mechanism is dislocation creep (i.e., power law creep). However, classic dislocation creep is not affected by grain size. Since grain size clearly must be considered based on the grain size difference found, it can be concluded that additional creep mechanisms are active beyond dislocation creep.

Internal defects caused by the PBF-LB/M process itself (such as porosity or cracks) can be excluded as reasoning for the creep behavior seen, since the relative density determined lies at 99.98%. The creep behavior of the three build orientations differs compared to the expected tensile behavior. Therefore, the material behavior seen in Figure 8, is assumed to be correlated specifically to creep deformation mechanisms.

It is known that grain orientation plays a significant role in accommodating the applied stress during creep. Therefore, Euler angles were determined based on EBSD-scans to assess the grain orientation with regard to the applied loading direction.

**Figure 9:**
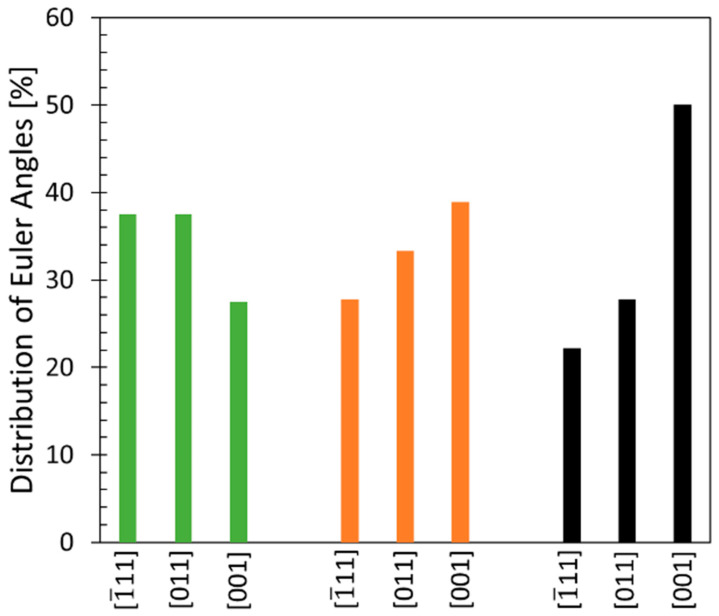
Euler angle distribution after creep testing for the 0°, 45° and 90° build orientation.

As can be seen in Figure 9, the 45° and 90° build orientation show a dominant orientation of [001], which is parallel to the applied loading direction. In the 0° build orientation, dominant Euler angles of [$\bar{1}$11] and [011] are seen. This explains the creep strains reached. Grains parallel to the load can accommodate the applied stress more readily than grains oriented perpendicular or diagonal to the load direction. The 0° build orientation show dominant orientations of [$\bar{1}$11] and [011] leading to inferior creep strains compared to the 45° and 90° build orientations, which both show dominant Euler angles in [001].

Although the 45° and 90° build orientations have a dominant Euler angle orientation in [001], the creep strains significantly differ. This can be attributed to additional creep mechanisms, which will be discussed later.

**Figure 10:**
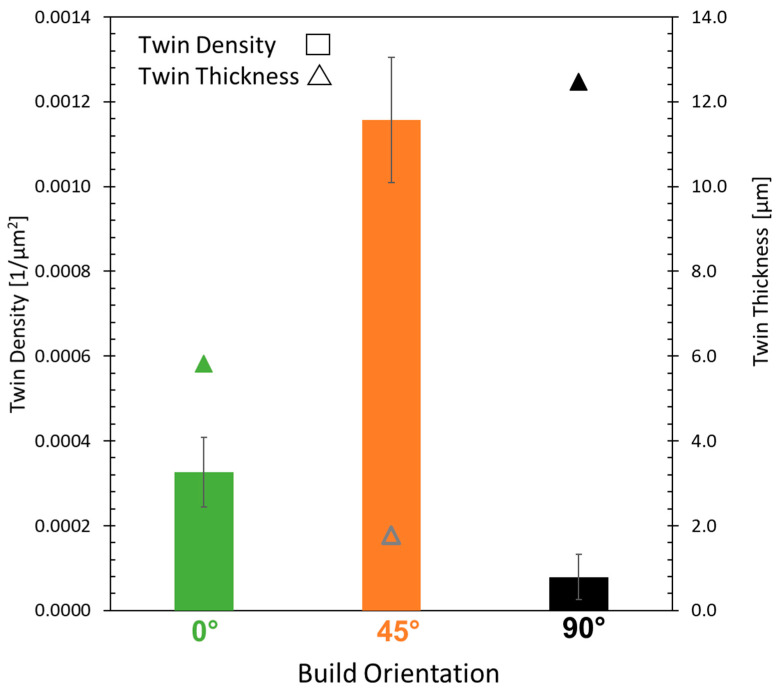
Twin density and twin thickness measured after creep testing for the 0°, 45° and 90° build orientation.

Twinning can be found in all build orientations. The twin density increases from 90° to 0° to 45° build orientation, as shown in Figure 10. Considering the creep rate documented in Figure 8, the twin density is inversely proportional to the creep rate. The twin density in Figure 10 combines annealing and mechanical twins. Since the heat treatment was identical for all build orientations, the number of annealing twins is assumed to be similar for all build orientations. The difference in twin density must therefore be caused by the formation of mechanical twins, which are formed during creep testing due to the applied mechanical load. Mechanical twins form mostly in hcp phases [40], which correlate well with the η-phase found in the 45° build orientation (refer to Figure 6).

Due to the differing solidification conditions experienced by the build orientations, Nb clusters were found (see Figure 5). As mentioned in the introduction, Nb atmospheres increase the formation of micro-twins during creep. Nb segregations have also been found to reduce twin thickness [41]. The determined twin thickness for all build orientations is plotted within Figure 10. The 45° build orientation, which contains Nb clusters, shows significantly thinner twins compared to the 0° and 90° build orientations, thereby indicating the formation of micro-twins. Besides Nb reducing twin thickness and increasing the formation of micro-twins, Nb segregations also lead to increased stacking faults energies (SFE) [13,17].

**Figure 11:**
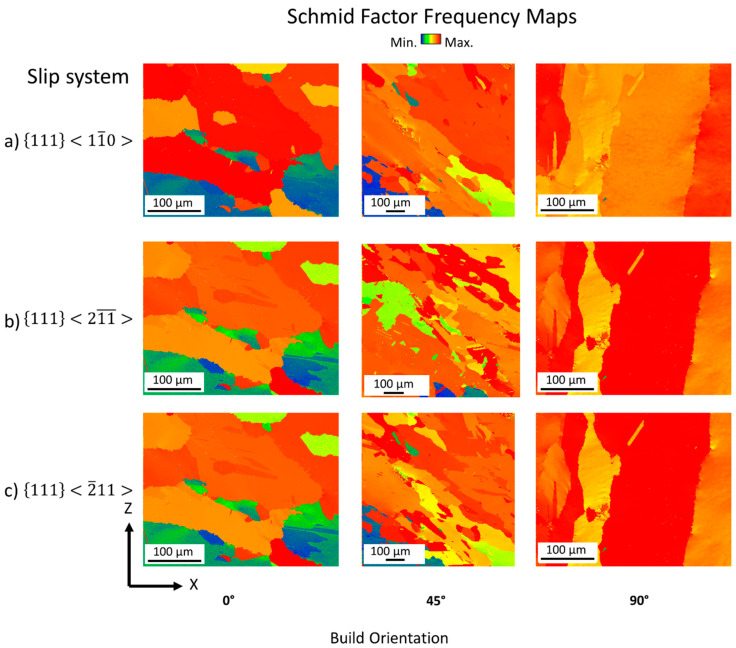
Schmid factor frequency maps for different slip systems (**a**) $\left\{111\right\}\langle 1\bar{1}0\rangle$; (**b**) $\left\{111\right\}\langle 2\bar{11}\rangle$; (**c**) $\left\{111\right\}\langle \bar{2}11\rangle$.

Since the Nb clusters reduce twin thickness and, thereby, the possible operating slip systems [41], Schmid factor frequency maps for three different slip systems were analyzed (see Figure 11):

- $\left\{111\right\}\langle 1\bar{1}0\rangle$ → fcc slip system
- $\left\{111\right\}\langle 2\bar{11}\rangle$ → slip system when Superlattice Intrinsic Stacking Faults (SISF) and/or micro-twins are present
- $\left\{111\right\}\langle \bar{2}11\rangle$ → slip system when Superlattice Extrinsic Stacking Faults (SESF) and/or micro-twins are present

Figure 11a shows the Schmid factor frequency for the slip system $\left\{111\right\}\langle 1\bar{1}0\rangle$ for all build orientations. The 90° sample shows a homogeneous activation of the fcc slip system, while the 0° and 45° build orientations show a non-uniform frequency of the fcc slip system. Figure 11b shows the results for the slip system $\left\{111\right\}\langle 2\bar{11}\rangle$. In the 0° sample, no significant change compared to Figure 11a can be seen. For the 90° sample still a homogenously activated frequency is identifiable. The 45° sample shows a more homogeneous frequency for the $\left\{111\right\}\langle 2\bar{11}\rangle$ slip system compared to the slip system shown in Figure 11a. The grains, in which a sudden change occurred for the $\left\{111\right\}\langle 1\bar{1}0\rangle$ slip system, show an activated Schmid factor for the slip system $\left\{111\right\}\langle 2\bar{11}\rangle$. This indicates that SISFs are present, as the slip system, $\left\{111\right\}\langle 2\bar{11}\rangle$, is closely linked to SISFs. Figure 11c shows the results for the slip system, $\left\{111\right\}\langle \bar{2}11\rangle$, which is closely linked to SESFs. The results for the 0° and 90° samples remain similar to those seen in Figure 11b. Similar to Figure 11b, in the 45° sample, the grains in which low Schmid factor frequencies are seen in Figure 11a and b are replaced by a higher Schmid factor frequency, as seen in Figure 11c. This indicates the presence of SISF (as seen in Figure 11b) and SESF (as seen in Figure 11c) in the 45° build orientation. As discussed in the introduction, the presence of SISF and SESF increases the SFE and increases the formation of micro-twins during creep. The increased SFE inhibits dislocation mobility. The energy required for dislocation slip in the 45° build orientation is therefore higher compared to twinning. This result is in agreement with that of Sanchez-Mata et al. [42] and explains the increased twin density in 45° compared to 0° and 90° build orientation found in Figure 10.

**Figure 12:**
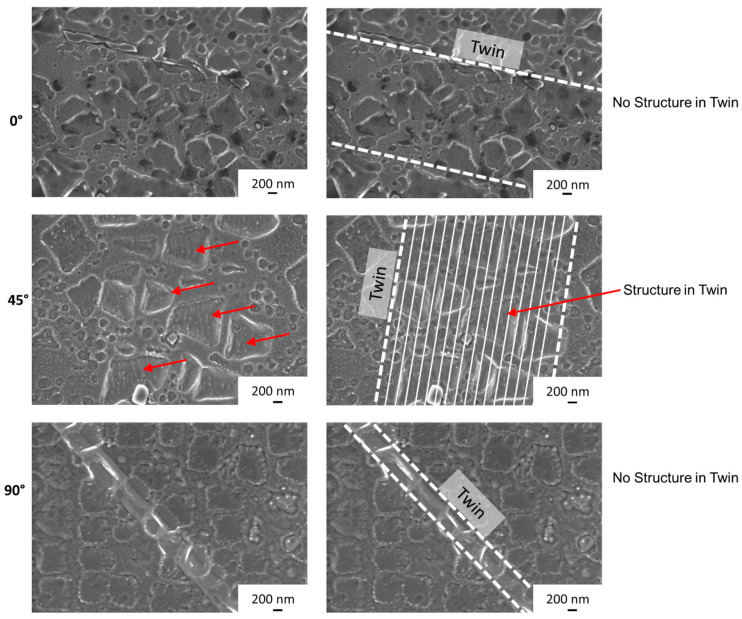
Twin structures for the 0°, 45° and 90° build orientations.

Based on the results above (Nb clusters, SESF, SISF) and the assumption of micro-twins, the twins present were investigated more closely (see Figure 12). Figure 12 shows representative images of the twins for the respective build orientations. On the right-hand side, the images are labelled for simplified visualization. Whereas the twins in the 0° and 90° build orientations do not show a structure, the 45° twins do show fine lines within the γ/$\gamma^{\prime }$ structure for the entire length of the twin.

**Figure 13:**
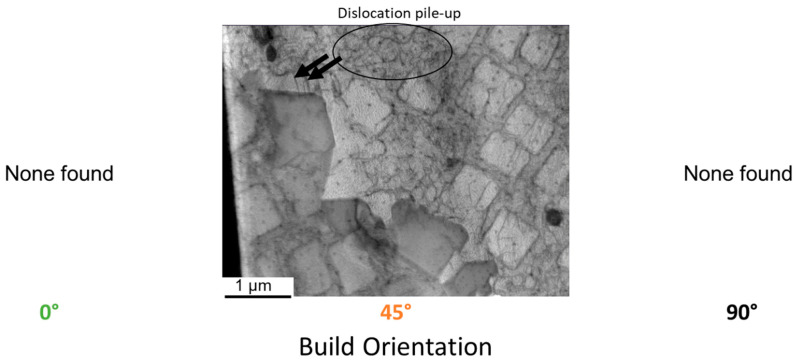
Micro-twins found in 45° build orientation.

The clusters of refractory metals, the expected increased stacking fault energy due to the presence of SISF and SESF, the Schmid factor frequency maps and the underlying lines within twins are all indications for micro-twins within the 45° build orientation. The macroscopic fracture surface of the 45° build orientation shown in Figure 8, is similar to those reported by Barba et al. for samples containing micro-twins [19]. TEM analysis was carried out to confirm these suspicions. Micro-twins were found for the 45° build orientation in the γ/$\gamma^{\prime }$ structure (see Figure 13). Close to the micro-twins a dislocation pile-up can be seen. Micro-twins were not found for the other two build orientations, 0°and 90°. The average thickness of the micro-twins is roughly 40 nm, which corresponds to 11 atomic planes, which fits into the micro-twin size range mentioned in the introduction. To the knowledge of the authors, this is the first time micro-twins are confirmed in additively manufactured samples.

Therefore, as expected, various creep deformation mechanisms are simultaneously taking place in the 45° build orientation: dislocation creep, micro-twinning, grain boundary sliding. The reason behind the differing active mechanisms between the build orientations (0°, 45° and 90°) can be found in the varying solidification conditions during PBF-LB/M. The difference in solidification conditions causes differences in the microstructure, including grain size, phase formation (carbides and η-phase) and elemental distribution. 

## 4. Conclusions

Within the scope of this study, the effect of three build orientations (0°, 45° and 90°) on PBF-LB/M creep properties was investigated. The following conclusions can be drawn based on microstructural investigations:

- Solidification conditions differ between the build orientations.
- The differing solidification conditions cause differences in grain size, phase formation and segregation behavior.
- Time to rupture is lowest in 45° compared to the 0° and 90° build orientations.
- The 45° build direction exhibits more creep deformation mechanisms compared to the 0° and 90° build directions.
- The differing solidification conditions affect elemental distribution, which in turn affect twin density, and twin thickness.
- The differentiating twin behavior causes an increase in SFE.
- Micro-twins were found for the first time in PBF-LB/M samples.

## Figures and Tables

**Table 2:** Sample dimensions and PBF-LB/M manufacturing details.

**Sample Dimensions**	Cylinders: Diameter = 13 mm Height = 80 mm
**PBF-LB/M machine**	EOS M290 (Krailling/Munich, Germany)
**Volume energy density**	69 J/mm^3^
**Layer thickness**	40 µm
**Heat treatment**	Held between 110–1230 °C under argon atmosphere Air cooling Held at 840 °C for 24 h under argon atmosphere Air cooling

## Data Availability

Data is not available.

## References

[1] (2016). Additive Fertigung_- Grundlagen_- Teil_3: Haupteigenschaften und Entsprechende Prüfverfahren (ISO_17296-3:2014).

[2] (2016). Additive Fertigung_- Grundlagen_- Teil_4: Überblick über die Datenverarbeitung (ISO_17296-4:2014).

[3] (2017). Additive Fertigung_- Grundlagen_- Terminologie (ISO/ASTM 52900:2015).

[4] Herderick E. (2011). Additive Manufacturing of Metals: A Review. Mater. Sci. Technol.Conf. Exhib..

[5] Diegel O., Nordin A., Motte D. (2019). A Practical Guide to Design for Additive Manufacturing.

[6] Sanchez S., Hyde C.J., Ashcroft I.A., Ravi G.A., Clare A.T. (2021). Multi-laser scan strategies for enhancing creep performance in LPBF. Addit. Manuf..

[7] Rickenbacher L., Etter T., Hövel S., Wegener K. (2013). High temperature material properties of IN738LC processed by selective laser melting (SLM) technology. Rapid Prototyp. J..

[8] Megahed S., Krämer K.M., Heinze C., Kontermann C., Udoh A., Weihe S., Oechsner M. (2023). Influence of build orientation on the creep behavior of IN738LC manufactured with laser powder bed fusion. Mater. Sci. Eng. A.

[9] Viswanathan G.B., Sarosi P.M., Whitis D.H., Mills M.J. (2005). Deformation mechanisms at intermediate creep temperatures in the Ni-base superalloy René 88 DT. Mater. Sci. Eng. A.

[10] Kovarik L., Unocic R.R., Li J., Sarosi P., Shen C., Wang Y., Mills M.J. (2009). Microtwinning and other shearing mechanisms at intermediate temperatures in Ni-based superalloys. Prog. Mater. Sci..

[11] Ghosh S., Weber G., Keshavarz S. (2016). Multiscale modeling of polycrystalline nickel-based superalloys accounting for subgrain microstructures. Mech. Res. Commun..

[12] Kear B.H., Oblak J.M. (1974). Deformation modes γ’precipitation hardened nickel-base alloys. J. Phys. Colloques.

[13] Barba D., Pedrazzini S., Vilalta-Clemente A., Wilkinson A.J., Moody M.P., Bagot P., Jérusalem A., Reed R.C. (2017). On the composition of microtwins in a single crystal nickel-based superalloy. Scr. Mater..

[14] Smith T.M., Unocic R.R., Deutchman H., Mills M.J. (2016). Creep deformation mechanism mapping in nickel base disk superalloys. Mater. High Temp..

[15] Freund L.P., Messé O.M., Barnard J.S., Göken M., Neumeier S., Rae C.M. (2017). Segregation assisted microtwinning during creep of a polycrystalline L12-hardened Co-base superalloy. Acta Mater..

[16] Smith T.M., Esser B.D., Antolin N., Viswanathan G.B., Hanlon T., Wessman A., Mourer D., Windl W., McComb D.W., Mills M.J. (2015). Segregation and η phase formation along stacking faults during creep at intermediate temperatures in a Ni-based superalloy. Acta Mater..

[17] Titus M.S., Mottura A., Babu Viswanathan G., Suzuki A., Mills M.J., Pollock T.M. (2015). High resolution energy dispersive spectroscopy mapping of planar defects in L12-containing Co-base superalloys. Acta Mater..

[18] Smith T.M., Esser B.D., Antolin N., Carlsson A., Williams R.E.A., Wessman A., Hanlon T., Fraser H.L., Windl W., McComb D.W. (2016). Phase transformation strengthening of high-temperature superalloys. Nat Commun.

[19] Barba D., Alabort E., Pedrazzini S., Collins D.M., Wilkinson A.J., Bagot P., Moody M.P., Atkinson C., Jérusalem A., Reed R.C. (2017). On the microtwinning mechanism in a single crystal superalloy. Acta Mater..

[20] Sanchez S., Gaspard G., Hyde C.J., Ashcroft I.A., Ravi G.A., Clare A.T. (2021). The creep behaviour of nickel alloy 718 manufactured by laser powder bed fusion. Mater. Des..

[21] Pegues J., Lee S., Jensen S., Saiz D., Whetten S., Kustas A., Shamsaei N. (2023). Tailoring microstructure and strength through selective remelting in laser powder bed fusion. Mater. Sci. Eng. A.

[22] (2019). Metallic Materials—Uniaxial Creep Testing in Tension—Method of Test (ISO 204:2018).

[23] (2018). Metallic Materials—Calibration and Verification of Static Uniaxial Testing Machines—Part 1: Tension/Compression Testing Machines—Calibration and Verification of the Force-Measuring System (ISO 7500-1:2018).

[24] Hu Y., Tang D., Yang L., Lin Y., Zhu C., Xiao J., Yan C., Shi Y. (2023). Multi-physics modeling for laser powder bed fusion process of NiTi shape memory alloy. J. Alloys Compd..

[25] Wolfer A.J., Aires J., Wheeler K., Delplanque J.-P., Rubenchik A., Anderson A., Khairallah S. (2019). Fast solution strategy for transient heat conduction for arbitrary scan paths in additive manufacturing. Addit. Manuf..

[26] Moran T.P., Warner D.H., Phan N. (2021). Scan-by-scan part-scale thermal modelling for defect prediction in metal additive manufacturing. Addit. Manuf..

[27] Liu B., Fang G., Lei L. (2021). An analytical model for rapid predicting molten pool geometry of selective laser melting (SLM). Appl. Math. Model..

[28] Duong E., Masseling L., Knaak C., Dionne P., Megahed M. (2022). Scan path resolved thermal modelling of LPBF. Addit. Manuf. Lett..

[29] Ji X., Wang Y., Liang S.Y. (2022). Analytical modeling of temperature evolution in laser powder bed fusion considering the size and shape of the build part. J. Mater. Process. Technol..

[30] Zielinski J., Theunissen J., Kruse H., Rittinghaus S., Schleifenbaum J.H., Zhu D., Megahed M. (2023). Understanding inhomogeneous mechanical properties in PBF-LB/M manufactured parts due to inhomogeneous macro temperature profiles based on process-inherent preheating. J. Manuf. Mater. Process..

[31] Inco, The Internactional Nickel Company, Inc Alloy IN-738 Technical Data. https://www.nickelinstitute.org/media/1709/in_738alloy_preliminarydata_497_.pdf.

[32] Aggarangsi P., Beuth J.L. Localized Preheating Approaches for Reducing Residual Stress in Additive Manufacturing. Proceedings of the 2006 International Solid Freeform Fabrication Symposium.

[33] Mercelis P., Kruth J.-P. (2006). Residual stresses in selective laser sintering and selective laser melting. Rapid Prototyp. J..

[34] Risse J. (2019). Additive Fertigung der Nickelbasis-Superlegierung IN738LC Mittels Selektivem Laserstrahlschmelzen.

[35] Kunze K., Etter T., Grässlin J., Shklover V. (2015). Texture, anisotropy in microstructure and mechanical properties of IN738LC alloy processed by selective laser melting (SLM). Mater. Sci. Eng. A.

[36] Wang H., Zhang X., Wang G.B., Shen J., Zhang G.Q., Li Y.P., Yan M. (2019). Selective laser melting of the hard-to-weld IN738LC superalloy: Efforts to mitigate defects and the resultant microstructural and mechanical properties. J. Alloys Compd..

[37] Choi B.G., Kim I.S., Kim D.H., Seo S.M., Jo C.Y. (2004). ETA Phase Formation During Thermal Exposure and Its Effect on Mechanical Properties in Ni-Base Superalloy GTD 111. Proceedings of the Superalloys 2004 (Tenth International Symposium).

[38] Liu G., Xiao X., Véron M., Birosca S. (2020). The nucleation and growth of η phase in nickel-based superalloy during long-term thermal exposure. Acta Mater..

[39] Bürgel R., Jürgen Maier H., Niendorf T. (2011). Handbuch Hochtemperatur-Werkstofftechnik.

[40] Callister W.D., Rethwisch D.G. (2015). Fundamentals of Materials Science and Engineering: An Integrated Approach.

[41] Egan A.J., Xue F., Rao Y., Sparks G., Marquis E., Ghazisaeidi M., Tin S., Mills M.J. (2022). Local Phase Transformation Strengthening at Microtwin Boundaries in Nickel-Based Superalloys. Acta Mater..

[42] Sanchez-Mata O., Wang X., Muñiz-Lerma J.A., Atabay S.E., Attarian Shandiz M., Brochu M. (2021). Dependence of mechanical properties on crystallographic orientation in nickel-based superalloy Hastelloy X fabricated by laser powder bed fusion. J. Alloys Compd..

